# Current Challenges and Future Trends of Enzymatic Paper-Based Point-of-Care Testing for Diabetes Mellitus Type 2

**DOI:** 10.3390/bios11120482

**Published:** 2021-11-27

**Authors:** Margarita Ortiz-Martínez, Raquel Flores-DelaToba, Mirna González-González, Marco Rito-Palomares

**Affiliations:** Tecnologico de Monterrey, Escuela de Medicina y Ciencias de la Salud, Monterrey 64710, NL, Mexico; margarita.ortiz.mtz@tec.mx (M.O.-M.); raquel.floresdlt@gmail.com (R.F.-D.)

**Keywords:** point-of-care testing, paper-based analytical device, colorimetry, glucose, type 2 diabetes mellitus

## Abstract

A point-of-care (POC) can be defined as an in vitro diagnostic test that can provide results within minutes. It has gained enormous attention as a promising tool for biomarkers detection and diagnosis, as well as for screening of chronic noncommunicable diseases such as diabetes mellitus. Diabetes mellitus type 2 is one of the metabolic disorders that has grown exponentially in recent years, becoming one of the greatest challenges to health systems. Early detection and accurate diagnosis of this disorder are essential to provide adequate treatments. However, efforts to reduce incidence should remain not only in these stages but in developing continuous monitoring strategies. Diabetes-monitoring tools must be accessible and affordable; thus, POC platforms are attractive, especially paper-based ones. Paper-based POCs are simple and portable, can use different matrixes, do not require highly trained staff, and are less expensive than other platforms. These advantages enhance the viability of its application in low-income countries and hard-to-reach zones. This review aims to present a critical summary of the main components required to create a sensitive and affordable enzymatic paper-based POC, as well as an oriented analysis to highlight the main limitations and challenges of current POC devices for diabetes type 2 monitoring and future research opportunities in the field.

## 1. Introduction

Detection of biomarkers is essential for early diagnosis and good disease management, but their low abundance and complex biological surrounding makes their detection and quantification challenging. Conventional methods for their determination usually require lengthy analysis times, expensive reagents, sophisticated equipment, and specialized personnel [1,2]. In this context, paper-based platforms are an attractive alternative for biomarker detection with broad advantages, as they are simple, robust, and cost-effective [1,3]. Paper-based point-of-care (POC) devices allow for reduced testing time and reagent volumes and do not require specialized equipment or personnel, making a large-scale screening strategy more feasible [4,5]. This renders them especially useful in remote communities and low-income countries where the budget, specialized personnel, and health infrastructure are not available to perform analytical methods such as mass spectrometry, chromatography, or immunological methods on a mass scale [1,6]. Moreover, their successful application has been reported for environmental [5], food [7,8], and clinical diagnostic analysis [9,10]. A POC test must offer clear advantages over traditional centralized laboratory testing regarding cost, convenience, or improved quality of care, equaling or exceeding sensitivity and accuracy requirements [11]. Furthermore, these considerations should be addressed starting with the early stages of prototype design.

Most reported paper-based platforms comply with the World Health Organization’s “ASSURED” (Affordable, Sensitive, Specific, User-friendly, Rapid and Robust, Equipment-free, and Deliverable to end-users) criteria, which are guidelines for the evaluation of diagnostic tests that any test designed for application in developing countries is recommended to fulfill [12,13,14]. The Special Programme for Research and Training in Tropical Diseases (TDR) originally proposed the ASSURED criteria for evaluating diagnostic tests for infectious diseases [14]. However, it has been extended as per the requirements for any POC for diagnostic purposes, especially those intended for low and middle-income countries.

Early detection and management are of particular relevance in chronic noncommunicable diseases (NCDs) due to the generation of complications that significantly impact the life quality of patients and can even lead to death. NCDs, particularly cancer and cardio-metabolic diseases such as cardiovascular disease, arterial hypertension, and type 2 diabetes (T2DM), are undoubtedly among the greatest challenges for health systems in low- and middle-income countries like Mexico [15,16]. T2DM is one of the fastest-growing global health emergencies of the 21st century. Currently, almost 500 million people live with diabetes in the world. It is estimated that by 2030 this number will reach 578 million and 700 million by 2045 [16]. T2DM is a chronic metabolic disease characterized by elevated blood glucose levels due to deficiency in insulin production and secretion in pancreatic β-cells, the development of insulin resistance in tissues, or a combination of both mechanisms [17,18]. The insufficiency of detection technologies accessible and cost-effective that adequately reflect glycemic changes has negatively impacted T2DM screening, diagnosis, and monitoring, particularly in low- and middle-income countries. 

Clinically validated paper-based platforms may be a starting point for developing continuous monitoring devices that can be added to existing wearables such as smartwatches [1,19]. This would be particularly relevant in T2DM because it is known that fluctuations in glycemic levels occurring at specific times during the day can lead to severe complications despite mean fasting glycemic values remaining in the normal range. 

Although multiple proofs-of-concept of POC platforms have been reported, and some of them have even been successfully evaluated with human samples for clinical validation, their market entry has not been achieved. These products require large-scale validation studies and regulatory approvals to enable their commercialization [19]. It is important to consider that the values of accuracy, sensitivity, and specificity obtained at the research level for the reported POC platforms should be taken as a valuable benchmark for choosing the best-performing systems for the analytical determination of the biomarkers of interest in order to guide the development and optimization of new platforms. However, it cannot be immediately transferred to the clinical setting. These parameters should be evaluated with greater scrutiny during the clinical validation process using real patient samples and under the standards of international organizations, comparing, if possible, against certified and standardized methods. The high cost, lengthy development time, and required regulatory filings are challenges to the market implementation of new POC devices; however, it is clear that new tools for screening, early diagnosis, and monitoring are needed to combat the current T2DM pandemic [10,14]. 

Paper-based devices are platforms with the potential to generate robust, sensitive methods that detect metabolic changes in the medium and short term that do not require specialized equipment or personnel, and they must be affordable to allow their large-scale use even in low-income and remote locations. Using biological fluids other than blood could generate non-invasive devices. The development of tools of this type could positively impact the incidence, improve management, and reduce the prevalence of underdiagnosis of T2DM. Furthermore, continuous monitoring devices and POCs bring us even closer to the goals of personalized medicine. However, it is essential to consider that there are still challenges and areas of opportunity for developing and implementing these platforms, which must be addressed to allow their successful entry into the market. This review aims to present a critical summary of the main components required to create a sensitive and affordable enzymatic paper-based POC, as well as an oriented analysis to highlight the main limitations and challenges of current POC devices for T2DM monitoring and future research opportunities in the field.

## 2. Main Components of Paper-Based Point-of-Care Platforms

There are several aspects to consider in developing paper-based POC platforms, from the technical aspects involved in their development and manufacture to obtaining the results qualitatively or quantitatively and their interpretation. These aspects depend primarily on the disease under study and the biomarker of interest, but other specific factors are also involved. The main considerations for developing paper-based platforms and their applications are described in the following subsections: platform design and fabrication, detection technologies, and applications.

### 2.1. Platform Design and Fabrication

Since their introduction, POC platforms have been widely implemented as additional tools for diagnosing or monitoring diseases as well as detecting molecules such as proteins, nucleic acids, metabolites, or microbes/pathogens. These tools can use various biological fluids (mainly blood, saliva, urine, sweat, and tears) as a matrix to detect a specific molecule or target. Therefore, design and fabrication of a POC platform depend on its objective, target, and sample selection [20]. Another factor to consider when designing and fabricating a POC is the end-user; the user can be the patient or workers of the clinical field, who are not necessarily highly trained personnel. On the other hand, the environment where the test will be performed plays an important role because it could be designed to be used at the patient’s home, in clinics, or in rural areas in low-middle income countries, where medical resources are limited [21]. Thus, these factors are part of the success of a POC test and should guide its manufacturing, but other factors may influence the development of this type of test. 

In the case of devices developed by universities or research centers, the feasibility of not publishing the initial stages of development, i.e., the technical specifications, should be assessed in order to protect the intellectual property associated with the devices and give priority to the publication of the evidence of their clinical performance in terms of sensitivity and specificity [11,22]. The time required to complete the approval process for a microfluidic-based device has been reported to vary from 4 to 14 years [11]. The ideal scenario to reduce these times would be to generate a critical path of the regulatory procedures necessary for the approval of the POC device for clinical use during the design stage. It would be advisable to consult the guidelines of internationally recognized bodies such as the FDA as well as the corresponding national regulatory authorities. However, it is important to note that regulatory procedures depend on the type of device and its intended use. There may be discrepancies between national and international regulatory authorities, which prevents generalized regulatory pathways that apply to any device. In some cases, specialized consulting may even be required to complete the approval process for these devices. In addition, the costs associated with these procedures must be considered in the initial investment required to bring these devices to market. 

Available POC tests can be categorized into several groups according to their practical use, type of platform, complexity, detection method, sample matrix, and readout, meaning the design and fabrication of POC may follow several paths [23]. One of the crucial publications driving the development of paper-based analytical platforms coupled to colorimetry for low-cost multi-metabolite analysis even in non-clinical settings was the 2007 paper by Dr. Whiteside’s group [24]. Most importantly, the work of this research group has focused its efforts on developing these novel platforms for diagnostic use to generate accessible and cost-effective tests whose design can take advantage of already clinically validated analytical methods [13]. Paper-based POC platforms are one of the most promising and widely researched platforms due to their simplicity, affordability, rapidity, and ease of use. The main component of this type of test is paper, and it has gained ground in its use as the substrate for POC testing. In addition to being affordable and accessible, paper is extremely versatile. There are different types of paper, but it is typically made of cellulose or cellulose-polymer blends, making it compatible with biological samples, so it can easily be used in the manufacture of POC tests [13]. Filter paper is frequently used for POC manufacturing; it works well for large molecules because of its well-defined pore size. Nitrocellulose membrane is usually employed for protein immobilization [25]. Paper modified with biomolecules can be used for pathogen detection in microfluidic paper-based analytical devices (µPADs) when simplicity is required. Cellulose *glossy* paper can also be used as a substrate for µPADs with the advantage that it allows the modification of its surface with nanoparticles [25]. There is a wide range of this type of test available, which can be classified into three main categories: paper test strips or dipsticks, lateral flow (LF) assays, and µPADs [21,26,27]. 

Paper test strips or dipsticks are a qualitative paper-based POC that generally consists of a chemically sensitive strip with a detection zone made of paper with immobilized reagents; hence, they are based on detection principles involving chemical-indicator reactions to immunological reactions, and the result is obtained by immersion and optical visualization. Therefore, dipsticks are used to detect several analytes that are easily found in biological fluids thanks to their nature. However, despite their simplicity, affordability, and availability, they exhibit some drawbacks such as inaccuracy, color change when the timing is exceeded, and subjective interpretations [23,25,26,27,28]. In order to improve the detection of targets, dipsticks may include the use of devices for reading the results (discussed later); in this way, detection becomes quantitative instead of qualitative [23].

On the other hand, LF POC are useful tools because of their rapidity and low cost. They mostly consist of nitrocellulose membranes in a chromatographic manner with control lines and a sample pad where the conjugate is stored. It involves the passive movement of the sample solution containing the target in the test area under the effects of capillarity, and then the absorption in the pad takes place along with a color change [25,29,30]. LF can be sub-classified based on its design and the type of target being analyzed. Regarding the design, there is the variation named two-dimensional paper networks (2DPN), as compared to LF, it extends its use to two dimensions, incorporating multi-step chemical processing and improving their detection limits. They use multiple fluid inputs that converge while controlling the time at which they reach the detection zone, and at the same time, the test maintains advantages such as simplicity and speed of LF POC test [30,31]. In terms of the type of target, LF can be divided into lateral flow immunoassays (LFIA), nucleic acid lateral flow assays (NALF), and nucleic lateral flow immunoassays (NALFIA). LFIA is based on proteins such as antibody-antigen interactions, NALF is based on the hybridization of two complementary DNA or RNA strands, and NALFIA uses nucleic acid or antibody–antigen, or both, as recognition elements [26,27]. Dipsticks and LF tests are excellent options as they are mainly affordable and simple to use, but these devices may suffer inadequate detection limits and restricted ability to provide quantitative measurements. New devices that address these limitations have been introduced and widely studied in the last decade, including paper-based POC tests using microfluidics [32]. µPADs consist of paper that allows hydrophobic/hydrophilic demarcations using various polymers, glass silicon, or paper as substrate and can be fabricated using two-dimensional or three-dimensional techniques, depending on the complexity of the application [25,28,33]. Unlike two-dimensional devices, three-dimensional devices shorten analysis times and enable the integration of multiple analyses and minimize sample evaporation due to the combination of lateral and vertical flows [34,35]. However, their manufacturing process is more complex and requires additional materials for assembly [36]. In addition, µPADs employ different fabrication procedures, which can be divided into 2D shaping/cutting and physical blocking of pores. µPAD can be sub-classified by physical and chemical techniques such as inkjet printing, ink stamping, wax printing, wax dipping, wax screen printing, paper cutting, shaping plasma treatment, laser treatment, photolithography, and chemical vapor-phase deposition, among others. However, almost every fabrication technique has a drawback like the need for manufacturing equipment, expensive instruments, and reagents or low resolution, limiting the introduction of this kind of POC to the market [25,28,33]. 

In addition to the platform’s design, the interpretation of POC results can be performed by eye or an external reader; thus, the readout can be carried out by applying distance-based, text-based, time-based, and smartphone-based methods. The distance-based readout is easy to interpret because it incorporates a scale for measurement or diagnosis. The text-based method provides semi-quantitative results directly on the test strips. The time-based method implies reading the result within a specific time frame. Moreover, POC tests can be coupled to smartphones and analyze testing results, giving quantitative information and strengthening its potential as a POC. However, it is necessary to standardize the conditions of use to ensure that the result can be read without interference [21,37]. 

**Figure 1:**
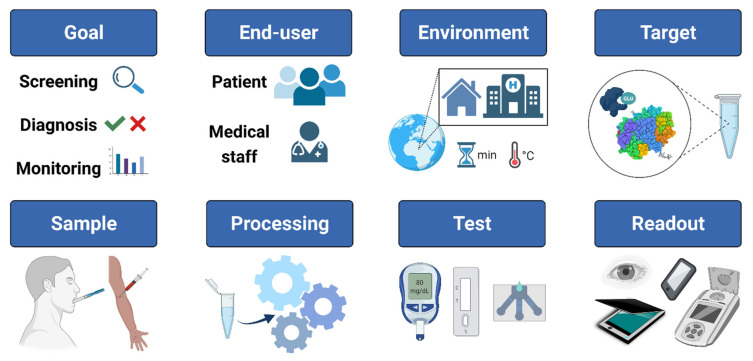
Principal factors involved in the design and manufacturing of point of care (POC) devices. Created with BioRender.

The POC platforms previously mentioned are the most used and studied, but other novel POC designs exist. However, these are considered more complex because they apply different analytical principles for detection such as spectrophotometric and enzyme-activity measurement, immunoassays, sensor-based blood-gas analysis, and hematological particle counting. In addition to the fact that they need to be coupled to other instruments that are not easily accessible, limiting their inclusion as POC, as they do not entirely fulfill the ASSURED criteria [23]. Detection, diagnostics, and monitoring tests have become widely available in the last few years as they are an effective tool in many different fields, including clinical. Although countless design, performance, and implementation reports are available, and a broad range of diagnostic devices such as POC are being developed, it is difficult to establish a guideline to make test selection easier [12]. Therefore, there is no exact guide on how to manufacture a paper-based POC. The factors mentioned above should be taken into account to carry out the design as proposed in Figure 1. 

The first consideration is the test’s purpose, i.e., if it will be used for screening, diagnosis, or monitoring of disease. According to the selected goal, it should be evaluated whether the patient or medical staff will perform the test. If the patient performs it, high sensitivity can be sacrificed, and a more straightforward technique such as dipstick or a LF device can be employed, but if it is performed by medical staff, simplicity and cost can be placed second, and more complex platforms such as some variant of µPADs can be used. In addition to the end-user, the test site is delimited by evaluating which detection method is most appropriate according to the requirements, such as the need for coupling external reading or naked-eye detection systems. One of the most critical points to consider is the target, as the nature of the biomarker plays a big role because its chemical or physical properties can determine the type of detection required. The sample and its processing involve factors such as sample availability, accessibility to sampling, and presence of interferents. Saliva is one of the most promising fluids for use in POC due to its availability, easy collection, the lack of need for highly trained personnel, and the fact that it has a great variety of metabolites in its composition that function as promising biomarkers for various diseases. Finally, defined by the previous factors, one can opt for any of the platforms mentioned before; dipsticks are efficient for single or large molecules, LF assays are suitable for proteins and nucleic acids, and µPADs can be more specific for different types of molecules. Each platform involves different detection methods (colorimetric, fluorescent, chemiluminescence/luminescence, or electrochemical), and the readout of the results can be coupled to different devices such as external handheld readers and smartphones or the naked eye.

The accelerated advancement in smartphone optical technology and their wide availability make them very attractive candidates for application in imaging and processing coupled to POC test platforms, especially those based on colorimetric methods. To exploit the capabilities of these devices, applications designed to detect signals and allow their processing on the same device, even allowing a preliminary analysis of the results, have been reported [1,38]. An example of this is “Glucosensing”, explicitly designed for glucose detection [39]. 3D printing can enable the design of holders to allow images to be captured with smartphones in a reproducible way, allowing detection to be carried out with greater accuracy [5].

Despite the emergence of novel methods and techniques for biomarker detection and biomarker panels in multiplex systems with high accuracy and sensitivity, they represent high operating costs and require specialized training, sophisticated equipment, and high volumes of expensive reagents. As a result, they are not widely adopted in the clinical setting in low- and middle-income countries. In the case of microfluidic systems, there is the benefit of miniaturization with a wide variety of design and manufacturing techniques, and these platforms seek to reduce reagent consumption without sacrificing high performance [40,41]. Paper-based POCs can achieve the potential for high-throughput screening by performing multiple reactions or multi-step screening even in low-cost settings and have been shown to be feasible to manufacture at low cost and with simple laboratory equipment [40,42]. For the high-throughput production of these devices, the use of automated equipment, paper pretreatments, and the search for new technologies and methods for more sensitive and accurate detection of biomarkers have been proposed, with the disadvantage that this increases design and manufacturing costs [43,44]. In a 2017 report, glucose determination was used to verify the performance of the high-throughput rapid-prototyping method for the fabrication of paper-based devices, and a linear relationship between concentration and color intensity was observed [43]. Additionally, the development of paper-based POC electrochemical devices for multiplex analysis has been reported [44,45]. Such platforms allow high-throughput analysis with large-scale throughput capability. As a proof of concept, this platform was used for the determination of glucose in urine successfully [44].

### 2.2. Detection Technologies

POC devices employ various methods to detect or quantify the specific target in order to obtain a test result. These methods should be designed for end-user interpretability, considering the environmental conditions. The selection of the detection technique could be performed after choosing the platform design, as it mostly applies to LF assays or µPADs. In the case of dipsticks, generally, there is no requirement for this selection as it is based on optical detection, but it can be applied if improvement with an external reader is desired. A wide range of detection techniques are available using different technologies, but the most commonly used are the colorimetric, fluorescent, chemiluminescence/luminescence, and electrochemical sensing methods. [21,28]. Some detection technologies are more suited to specific platforms because of their nature, the type of paper used, and the selection of a specific molecule as the target. Regardless of which one of these technologies is used, the result should be presented without additional interpretation. Next, the characteristics of the most popular detection methods are highlighted, including their advantages and limitations. 

#### 2.2.1. Colorimetric

The colorimetric method is mostly based on the reaction produced by specific reagents with the molecule of interest, which generates a detectable color change. This technique is based on redox, immunological, or enzymatic reactions, and when used on paper-based platforms, the detection involves the movement of an analyte solution in the test zone through capillarity [28]. Colorimetric detection is commonly applied in paper-based POC tests, given its multiple advantages associated with its being a low-cost and straightforward technique. These include avoiding the use of sophisticated equipment for reading results, having no need for highly trained personnel, and having relatively fast detection time as results are obtained in minutes to a couple of hours. All of these advantages provide feasibility for applications in remote areas [46,47]. Nevertheless, there are also certain limitations as results can be compromised when the test is not performed under specific parameters (specific readout time, temperature, and humidity). Therefore, the use of adequate color indicators is crucial in order to avoid a decrease in the sensitivity of the method [21,48]. Furthermore, this technique is qualitative and may be limited to yes-or-no results, but if the intensity of the color in the test area is a function of the concentration of the target, it is possible to measure the intensity with a camera or a smartphone, translating this information into concentration [13].

#### 2.2.2. Fluorescent

Fluorescence detection involves the interaction between target molecules and fluorophores. It involves three phases: (1) excitation, (2) excited-stated lifetime, and (3) fluorescence emission. In addition, the process requires a light source, optical sensors, optical alignment devices, and a signal processing unit. The light resulting from the process is filtered and quantified and is equivalent to the target concentration [28,48]. Different types of samples are compatible with this method, and it also provides higher sensitivity in results, yielding quantitative values. However, the limitation is the necessity for additional complex and expensive instruments [33,49]. Although this detection technique provides good concentration sensitivity, some studies have reported deviation between replicates and problems improving the precision of the measurements due to scattering of light on the cellulose fibers [50]. 

#### 2.2.3. Chemiluminescence and Luminescence

Chemiluminescence is based on the emission of light generated when a chemical reaction occurs between two reactants and a catalyst or excited intermediate. In contrast, luminescence is the light emission that appears when an excited molecule relaxes to its basal state [33,50]. The reagents commonly used are luminol and peroxidases; luminescent reagents more selective than luminol are available on the market, but these can be expensive. Additionally, chemiluminescence and luminescence achieve very low detection limits providing high sensitivity and are robust techniques for biomarker detection. However, the reagents that work based on redox reactions are strongly influenced by oxidants and antioxidants in the sample. Therefore, its high sensitivity could be a drawback, but this can be solved with the addition of sample processing by removing potential interferents [28,33]. Despite the clear advantages of this technique, it requires a separate instrument to measure the light emission, since these tests cannot be visualized with the naked eye because the emission is too low to detect changes without equipment, which are already on the market in miniaturized form but are not commercially available and are generally of very low sensitivity [51].

#### 2.2.4. Electrochemical

This is a surface technique based on electrochemical sensors, and it uses three main electrodes: (1) a counter electrode, (2) a working electrode, and (3) one or multiple reference electrodes. It employs electrochemical techniques for detecting results such as cyclic voltammetry, amperometry, coulometry, or potentiometry [33,50]. It is characterized by its low cost, portability, high selectivity, low electrical power consumption, fast responses, and high sensitivity. In addition to being independent on ambient light, it is less prone to changes or deterioration in the device if paper is used as support. The electrochemical detection devices also present the possibility of determining two or more targets providing a multiplex analysis. However, it involves various equipment or electrochemical elements and techniques, increasing its design and application complexity [28,50,52].

### 2.3. Applications

POC tests have gained enormous attention in the last few years due to their characteristics such as simplicity, portability, reduced costs in diagnosis, shorter waiting times, and not requiring highly trained staff or very specialized equipment. Additionally, most of these kinds of tests are designed to meet the majority of the ASSURED criteria. POC devices have been widespread in different fields, but the ones addressed to healthcare are of greatest interest because they are useful as an auxiliary in detecting diseases, specific targets such as biomarkers, and even microbes or pathogens. This translates into less waiting time, more accessible diagnostics, and especially a sustainable alternative tool for complex biomarker detection. For example, the determination of the presence or the exact concentration of common drugs with POC represents a useful tool in emergencies such as active bleeding, urgent surgery, and drug overdose. These tests provide valuable information for decision-making in record time. Continuous monitoring of these kinds of analytes can prevent overdosing when there is the need for periodical administration of some drugs, thus providing quality information to healthcare workers to define appropriate dosage [53,54]. Another important field where POCs have gained significance is in the auxiliary detection of infectious agents. Several tests available on the market can detect infectious pathogens and are mainly based on immunochromatography of a specific microbial antigen in a patient sample. This facilitates decision-making in the diagnosis and treatment of the patient [23]. Monitoring or detecting biomarkers (genes, proteins, lipids, or metabolites present in tumor tissues, serum, or body fluids) is critical for diagnosing multiple diseases, especially those associated with global health problems due to the current lifestyle of the world population. A biomarker can work as an indicator to differentiate abnormal stages of disease; thus, the use of POC testing for biomarker analysis helps measure the risks of disease complications that may develop as the disease progresses. This means that if patients have access to POC testing to monitor the biomarker(s) of relevance to their disease, it can increase the success rates of treatments [1].

## 3. Paper-Based Point-of-Care Platforms for Screening and Monitoring of Type 2 Diabetes Mellitus

Glucose is a six-carbon sugar, one of the most abundant in nature, and is the central element of human energy metabolism. Moreover, it is the main marker for the diagnosis of diabetes [55,56]. Glucose is an excellent model marker for developing paper-based POC devices due to its price, accessibility, ease of handling, lack of toxicity, relative chemical stability, high water solubility, and presence in relevant concentrations in various biological fluids [56,57]. Blood is the fluid of choice for the determination of biomarkers, and glucose is no exception. In healthy individuals, the reference range for fasting blood glucose is 70–100 mg/dL [58,59]. Values below and above the reference range are relevant to health status, with low values being considered hypoglycemia and above being considered hyperglycemia. When values are greater than 125 mg/dL in two or more tests, it is possible to diagnose diabetes [58,59]. The intermediate stage with values of 100 to 125 mg/dL is defined as prediabetes and is a high-risk state for the development of T2DM [58,60]. The methods for blood glucose determination are well established, calibrated, and automatized, but sample collection is invasive, uncomfortable, and potentially painful. Most are enzyme-based, especially those based on glucose oxidase (GOD) with detection by colorimetry or electrochemistry [56,61]. The first generation of glucose biosensors is based on the production and detection of peroxide with oxygen as a cosubstrate (Equation (1)). This reaction involves the reduction of the flavin group (FAD) in the enzyme to generate the reduced form of the enzyme (FADH2) (Equation (2)) [62,63,64].

(1) $$\mathrm{Glucose}+{\;O}_{2\;}\to \mathrm{gluconic}\;\mathrm{acid}+{\;H}_{2}O_{2}$$

(2) $$\mathrm{GOx}\;\left(\mathrm{FAD}\right)+\mathrm{glucose}\;\to \mathrm{GOx}\;\left({\mathrm{FADH}}_{2}\right)+\mathrm{gluconolactone}$$

These sensors have the disadvantage of being affected by electroactive interference, so some metabolites of interest and components of biological fluids can affect the selectivity of this type of sensor. Additionally, they can give erroneous readings due to fluctuations in oxygen availability [62,63,64]. The second generation of glucose biosensors was achieved by replacing oxygen with a synthetic electron acceptor, acting as a mediator transporting electrons. Examples of these mediators are ferrocene-derived compounds, conductive organic salts, quinone compounds, and transition-metal complexes. In the third generation of glucose biosensors, electrons are directly transferred between the enzyme and the electrode without mediators. This type of biosensor has allowed the generation of devices for continuous in vivo glucose monitoring [62,63,64]. The fourth generation of glucose biosensors comprises sensors based on metal nanostructures where glucose oxidation occurs directly on the electrode surface and does not require enzymes [65,66]. A more detailed analysis of non-enzymatic sensors, especially those based on electrochemistry for glucose determination, can be found in the review by Professor Wang et al. [67]. Despite the undeniable progress that fourth-generation electrochemical devices represent, it is important to note that the use of nanomaterials for the fabrication of non-enzymatic devices increases the cost and complexity of the manufacturing processes, hindering their development and implementation in countries with limited resources. In contrast, enzyme-based devices, specifically those employing colorimetric detection, have attracted attention for detecting glucose in biological fluids due to their low development cost and versatility, making them of particular interest to countries with limited resources and will be the focus of the following discussion. Colorimetry is the most reported technique for determining glucose in clinical samples, mainly through the bienzymatic system consisting of glucose oxidase (GOD) and horseradish peroxidase (HRP) coupled to chromogens. The reaction catalyzed by glucose oxidase results in the production of gluconic acid and hydrogen peroxide. Peroxidase catalyzes the reaction of the hydrogen peroxide with the chromogen(s) to generate the color change. The two most commonly used HRP chromogenic substrates are 4-amino antipyrine (4-AAP) and 3,3’,5,5’-tetramethylbenzidine (TMB) [55,57,68,69]. Examples of the widespread application of the bienzymatic system GOD/HRP are shown in Table 1. It is vital to choose the right chromogen during the design of the paper-based assay platform to achieve suitable selectivity and specificity values for clinical application. There are several reports of using 4-AAP as a chromogen with detection limits relevant not only for the glucose determination in blood [4,45,70,71,72,73] but also in other biological fluids with low concentrations such as tears [74], urine [73], and saliva [75,76]. Other systems, such as GOD-HRP-TMB [77] and GOD-HRP-o-dianisidine [78], have shown promising results for the detection of glucose in sweat. 

In their 2017 report, Kang et al. reported using a paper platform made of cellulose filter paper to determine glucose in tears by colorimetry [74]. Due to the low glucose concentration in tears, it is crucial to have a preconcentration step before the determination. The designed strip makes direct sampling possible due to its biocompatibility, and the printed wax barriers keep the reaction zone isolated from the sampling zone [74]. This study demonstrated the detection of glucose in clinically relevant ranges. However, although they report that the color change allows differentiation between diabetic and normoglycemic patient samples, both with the naked eye and by optical density, they did not evaluate this quantitatively. Therefore, they did not report the detection limit, sensitivity, or specificity. In 2018, another group reported the preparation of two devices, a µPAD and a Schirmer strip, according to the methodology reported by Kang et al. [74] but with the use of a gold complex encapsulated in a carbopol gel to detect without chromogens [79]. The µPAD was evaluated for the determination of blood glucose, and good performance for glucose selectivity and high reproducibility were observed, showing a strong linear correlation with the values obtained with a commercial glucometer [79]. A study with a larger sample size would allow evaluating its potential to discriminate diabetic patients based on its sensitivity and specificity. The Schirmer strip treated with the gold complex was evaluated in simulated tear fluid, where a linear response between luminescence intensity and glucose concentration was observed [79]. Further studies will be of great relevance to demonstrate the application of such platforms with real patient samples and their clinical validation comparing tear samples from diabetic and normoglycemic patients.

The GOD/ HRP system coupled to potassium iodide (KI) or TMB was employed for blood glucose detection, the POC platform was generated with the wax printing method using Whatman No. 1 paper as a support [82]. In order to diminish the effect of ambient light on color detection, a stand with controlled illumination was designed on which to place the smartphone [82]. One of the main factors affecting microfluidic platforms is the sample volume variation, which is a challenge that needs to be overcome to achieve the commercial application of a POC platform, since it would require users to introduce a standard amount of sample. In this paper, a comparison was made between a volume-independent platform (VI-µPAD) and a conventional platform (C-µPAD). In the conventional platform, it is observed that the color intensity increases with higher sample volume, even though the glucose concentration remains constant [82]. In the proposed VI-µPAD platform, the sample comes in direct contact with the enzymes and chromogen, and it is the colored product that travels to the detection zone, allowing a homogeneous and uniform color intensity. Moreover, this color is more related to the glucose concentration than to the sample volume. In addition, the use of TMB instead of KI for detection allows the detection limit to be significantly lower, without sacrificing its broad linear range (0–22 mM), which would allow its use for real samples with clinically relevant values [82]. In 2020, a similar wax-printed, chitosan-treated paper system sealed with lamination film to configure the µPAD was reported [70]. This µPAD employs a system based on peroxide generation by GOD and lactate oxidase (LOD) enzymes and its subsequent colorimetric detection by the HRP/4-AAP/DHBS system, and it includes a separation membrane to allow its use on blood samples directly without the need for pretreatment, and the color change detection was performed by capturing the images with a scanner. This system showed the ability to accurately detect glucose in serum and whole blood with high linearity and recovery rates in the range of 90–110%. The same research group reported a proof-of-concept of a similar platform for the simultaneous determination of glucose and lactate [4]. This system showed good selectivity; additionally, no significant color generation was observed when using interfering solutions (fructose, lactose, sucrose, NaCl, MgCl_2_, CaCl_2_, L-cysteine, and uric acid) as sample. The high selectivity for the biomarkers, conferred by the catalytic properties of GOD and LOD, would allow the use of this platform with serum samples [4]. In this system, it was possible to obtain a color change distinguishable to the naked eye for both analytes, and in addition, this µPAD has self-calibration capabilities. Furthermore, in the future, by incorporating a smartphone, it would be possible to move from a semi-quantitative to a quantitative detection. 

The GOD-catalyzed reaction of glucose to generate gluconic acid causes a pH change in the medium that can be detected using a pH indicator. Examples of such systems using bromocresol purple as an indicator have been reported, and these systems were able to determine glucose in saliva with high sensitivity and accuracy [75,81,85]. In their *2015* study, this research group reported a proof-of-concept using methyl red as a pH indicator and an office scanner as the device to acquire the color signal. However, despite showing potential in clinical ranges, this platform showed a high LOD of 22.2 mg/dL and was strongly affected by interferents commonly present in the samples, such as lactic and ascorbic acids [85]. In their 2017 report [75], they employed purple bromocresol as a pH indicator and a smartphone as a platform for color data acquisition. While in their subsequent work in 2019 [81], they reported using a standalone electronic meter, which avoids variability due to ambient light conditions. This device was validated with clinical saliva samples, and its performance was compared against blood glucose values measured with a conventional glucometer. In addition, a high correlation was observed between blood glucose and saliva glucose values of diabetic patients [81]. Other methods and approaches that have been evaluated for the generation of platforms of this type are substrate treatment with UV resins [72], organosilanes [45], and the coupling of GOD and 4-AAP with nanoparticles [73]. The generation of analytical platforms that do not require an electronic readout device, i.e., naked eye determinations, has been explored [86]. In a 2018 report, detection by the naked eye was evaluated on a paper platform with hydrophobic lanes generated by patterning with paraffin [76]. A chitosan treatment on the substrate improved the distribution of the reagents, generating a more homogeneous color reaction and increasing the material’s biocompatibility with the GOD/HRP bienzymatic system. In addition, this system was evaluated on saliva samples where it was shown to be accurate with recovery rates of 92 to 114% and low operator variation [76]. The color change obtained can be used to construct a semi-quantitative scale to determine glucose levels with the naked eye, similarly to urine test strips. The specific design features of a POC platform should be evaluated based on the biomarker and the intended use. Systems that generate qualitative or semi-quantitative results can be used for monitoring already diagnosed patients or screening patients with high-risk profiles, which can be enhanced with the generation of simple, portable, and even readable devices. In contrast, more accurate systems that generate quantitative results may be reserved for diagnosis and use in clinical settings where portability can be allowed to be reduced to some extent to accommodate more sophisticated reading methods. Colorimetry-based POC devices have been shown to be a viable alternative for method development for either of these approaches.

**Figure 2:**
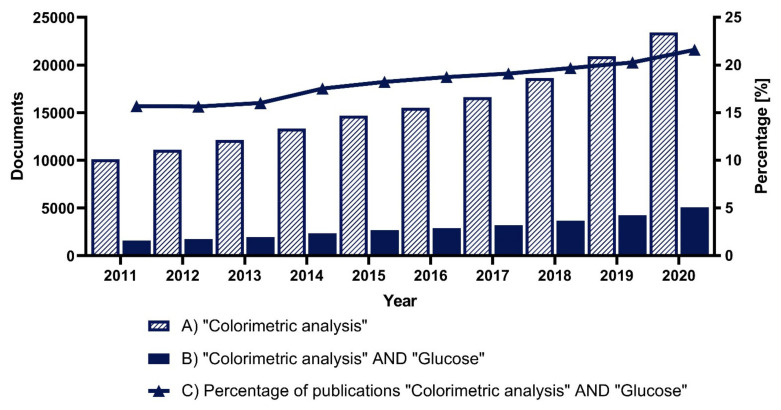
Analysis of the number of documents in Scopus over the last ten years: (**A**) using the search term “Colorimetric analysis”, (**B**) using the search terms “Colorimetric analysis” AND “Glucose”, (**C**) percentage of papers found using the term “Glucose” in the category “Colorimetric analysis”.

Despite the development of new technologies for glucose detection, interest in the use of colorimetry for glucose determination has not diminished in recent years [87]. This interest is evidenced by the steady increase in the number of publications on the subject in the last ten years, presented in Figure 2.

Since the recommendation made in 2009 by the International Expert Committee regarding glycated hemoglobin (HbA1c) as a long-term glycemic marker, this biomarker has been incorporated into worldwide clinical guidelines as a fundamental test for screening, monitoring, and diagnosis of T2DM [88,89,90]. One of the limitations of this biomarker is that the test must be carried out by a standardized and certified method to ensure the validity of the results. This standardization has been achieved in the United States and other parts of the world thanks to the National Glycohemoglobin Standardization Program (NGSP) and the Diabetes Control and Complications Trial (DCCT) assay [91,92]. Although POC devices for determining HbA1c are already on the market, these analyzers require a specific setting that limits their use outside clinical facilities, and their cost is not accessible, so the balance between portability, cost, and accuracy has not yet been achieved [93]. Several reports have evaluated the performance of POC analyzers compared to tests routinely employed in clinical laboratories. For example, in a meta-analysis published in 2017 [94], thirteen devices were evaluated, A1cgear, A1cNow, Afinion, B-analyst, Clover, Cobas b101, DCA 2000/Vantage, HemoCue, Innovastar, Nycocard, Quo-Lab, Quo-Test, and SDA1cCare. Nine of these devices showed a negative bias and large standard deviations, negatively affecting disease management [94]. In another study, the AfinionTM AS100 (Axis-Shield, Oslo Norway) and DCA VantageTM (Siemens Healthcare Diagnostics, Tarrytown, NY, US) analyzers were evaluated in comparison to conventional HPLC, both showing a good correlation with the conventional method [93]. However, both analyzers reported significantly lower values [93]. Subsequent studies have shown that analyzers have improved their performance [95,96,97]. However, some still present differences compared to conventional methods and should be used with caution in patients with renal failure. Moreover, the fact that they still require to be implemented in controlled settings prevents the development of a portable and accessible POC with its full potential [95,96,97]. Electrochemical microfluidic devices for HbA1c determination have also been reported [44,98,99]. Specifically, electrochemical impedance spectroscopy (EIS) has attracted attention for being a non-destructive and very sensitive biosensing technique. A three-dimensional paper-based device with EIS detection for the simultaneous determination of total hemoglobin and HbA1c was reported, showing high sensitivity for both analytes in ranges of clinical interest and a detection limit of 0.21% for HbA1c [44]. A nanobiosensor with a three-dimensional gold structure has also been documented for the determination of HbA1c in blood. Although it possesses the desirable characteristics of high sensitivity (269.2 mA/cm^2^) and low detection limit (0.0068 mg/dL), the concentration of HbA1c in blood is above the linear range of the biosensor, requiring sample dilution [99]. Undoubtedly, the use of nanomaterials to develop paper-based devices with electrochemical detection has driven the advancement of HbA1c determination. Thus, this fourth generation of biosensors represents an undeniable potential in the area of POC for diabetes screening and diagnosis.

Glycosylated albumin (GA) is another emerging biomarker for the screening and diagnosis of diabetes [100,101]. The most exploited methods for isolating and quantifying GA at clinical scale are affinity chromatography and enzymatic assays. One of its differential characteristics is that it has an intermediate detection range (2–3 weeks), and in some populations, it has shown a better performance than HbA1c for monitoring glycemic levels [101,102,103,104]. Despite the relevance of GA as a glycemic biomarker, there are still no commercially available POC devices for its determination. Nevertheless, it is expected that due to its potential and the high incidence of T2DM, advances will be soon emerging [105]. The development of microfluidic platforms for GA determination has benefited from nanomaterials that eliminate the need for the use of chromogens and enzymes. This technology has been used to develop a dipstick for GA determination achieving a detection limit of 6.59 μM in buffer and 8.7 μM in bovine serum [106]. The use of enzymatic processes has proven to be an area of interest for developing analytical platforms for disease monitoring and diagnosis. Currently, commercial kits are available to determine glycemic markers such as glucose, GA, and fructosamines (FAs). It is possible to use a clinically validated enzymatic method as a basis for developing POC devices, optimizing them for portability, and avoiding the need for a clinical laboratory [107]. A 2017 paper [107] reported an electrochemical sensor based on the coupling of an enzymatic method with a screen-printed carbon electrode for GA detection that could be used to develop a POC platform. This same research group reported the development of an enzyme-based electrochemical sensor, but this time they used an interdigitated electrode that allowed them to improve the sensitivity (2.8 nA/µM) and detection limit (1.2 µM) concerning their previous work [108]. Paper-based platforms can exploit their capabilities to generate multiplex assays, as in the case of a paper published in 2020 [71], which reports the simultaneous determination of hemoglobin, GA, and glucose on a paper-based platform with colorimetric detection. This platform showed detection limits of 0.23 mg/dL, 49.16 ng/mL, and 8.36 μg/mL for glucose, albumin, and GA, respectively [71]. 

FAs are a by-product of serum protein glycosylation that can serve as a marker of glycemic level [109,110,111]. This marker, like GA, represents an intermediate monitoring marker (2–3 weeks). Although commercial kits for FAs determination are available in some countries, their use in the clinic is limited [112,113]. The development of POC devices for FAs determination has not generated as much interest as other glycemic markers mentioned above. The development of a paper-based microfluidic platform using a wax-dipping process has been reported [111]. This platform allowed the determination of FAs corrected for variation in serum albumin by colorimetry using whole blood as a sample, with a membrane attached to the device for plasma separation [111]. Despite the advances in these biomarkers, glucose continues to be one of the most studied as a model molecule for the development of sensors and POC devices. In addition, the devices for its determination are among the most advanced not only in the management of diabetes, but in general in the diagnostic area, it is also one of the only biomarkers with continuous monitoring devices clinically validated and available on the market [105].

As the burden of diabetes grows worldwide and is especially critical for resource-limited countries, there is a growing interest in cost-effective alternatives for the screening and early diagnosis of T2DM [55]. For a novel platform to be accepted, the users’ point of view must be considered. The test should be easy to use, affordable, painless, and non-invasive, it should not require expensive or hard-to-maintain equipment, and it should present the results in a way that is to interpret [57]. There is currently a growing interest in developing novel, sensitive, accurate, rapid, and cost-effective methods for glucose detection. Paper-based POCs are an excellent alternative for conventional lab testing in T2DM because, in addition to meeting all these requirements, they have advantages such as portability and minimal sample consumption [55]. In addition, by using non-conventional sample fluids such as tears, sweat, or saliva, it would be possible to develop non-invasive platforms, which offer a competitive advantage in the market against traditional tests. Paper-based platforms have proven to be excellent alternatives for developing POC tests, and as mentioned in this review, their use in conjunction with colorimetric analysis has obvious advantages and benefits. However, one of their areas of opportunity is the limit of detection, which may prevent their application in non-conventional fluids. To overcome this challenge, other detection approaches have been analyzed, such as detection by electrochemical methods [114,115], distance-based [83,116], luminescence [79,117], fluorescence [79,118], calorimetry [119,120], and mass spectra [121,122]. Most of the paper-based POCs reported in Table 1 of this review have reported stability under refrigeration (4 °C) [4,69,73,76,79,80,84,123]. However, it would be better to ensure that the devices retain acceptable stability and low variability at different environmental conditions for mass implementation in screening programs.

## 4. Current Challenges and Future Trends

Since the turn of the century, the focus in the diagnostic field has been on developing continuous monitoring devices. Recently, the field has been fueled by advances in nanotechnology, advanced materials, and biosensors, focusing on the generation of wearables for continuous monitoring [19,124]. Recent advances in the development of microfluidic platforms and specifically epidermal electronics have demonstrated their potential in the area of continuous monitoring [125,126,127]. The application of epidermal electronics to generate minimally invasive biosensors for continuous glucose monitoring via microneedles has shown promising results in terms of sensitivity, specificity, selectivity, and response time [128,129]. Furthermore, this takes on a new level of relevance when considering their synergy with the internet of things (IoT), artificial intelligence, and big data analytics to enable real-time monitoring and computer-aided diagnosis [125]. 

The relevance of POC devices for the diagnosis of infectious diseases has been highlighted during the COVID-19 pandemic. It has been recognized that having this type of tool can positively impact the management of infectious diseases and could help in the prevention of epidemics. It would also allow the control of these epidemics in areas with low health service coverage, where traditional techniques are limited [130,131,132]. For nucleic acid detection, efforts will be expected to focus on developing techniques to replace classical PCR, such as isothermal amplification, with the advantages of allowing their inclusion in POC devices that meet the ASSURED criteria [132,133]. In 2021 [131], a report described the development of a paper-based POC device for detecting pathogen nucleic acids in saliva using loop-mediated isothermal amplification with the generation of a colorimetric response. This device demonstrated its ability to detect the SARS-CoV-2 virus without requiring sample pretreatment [131].

The design and manufacturing of paper-based devices have the great advantage of not requiring complex equipment or personnel with specialized training, which allows the generation of prototypes at a relatively low cost even in the same middle and low-income countries where their implementation is planned [134]. The global market for paper-based devices was estimated at 5.69 billion dollars in 2017, with a compound annual growth rate of 8% expected. Part of the impetus for this field comes from investments in research and development by government agencies such as the European Diagnostic Manufacturers Association and the growing interest of governments in affordable healthcare systems [135]. Bringing the new devices from the prototype stage to commercialization requires not only overcoming technical challenges and limitations but also the cooperation of governments, international agencies, and non-governmental organizations [136,137]. Examples of these international efforts by non-governmental organizations to support the development and implementation of new technologies, and diagnostic platforms are the programs developed by PATH (https://www.path.org/diabetes/, accessed on 22 May 2021), FIND (https://www.finddx.org/goals/, accessed on 22 May 2021), and the TDR (https://tdr.who.int/, accessed on 22 May 2021). The endeavor to successfully bring devices to market must be tackled with a multidisciplinary and even multi-institutional approach, involving universities, government agencies, and private organizations.

Some of the challenges that still need to be addressed in the generation and implementation of paper-based POC platforms are low reproducibility, high detection limits, low specificity, poor shelf life, and subjective interpretation of data [25,32,133,138]. While some microfluidic platforms have been reported to address most of these challenges, many of them sacrifice the low-cost or ease-of-use required to meet ASSURED criteria. In addition, their design and implementation take longer and increase their production cost by increasing their complexity [32,133,138]. Paper-based devices lack the mechanical robustness of materials used in the design of other multifluidic platforms such as glass, polymers, and silicon [42]. For selecting the patterning method for mass production of paper-based POC, performance, ease of use, and cost of manufacture should be considered, as some of the reported methods, such as stamping, are not suitable for mass production [43,139]. Colorimetric detection coupled with paper-based POC devices may have disadvantages such as low fluid distribution homogeneity and color formation homogeneity [40,84]. Although paper-based POC platforms with colorimetric detection present variations when detection is carried out with the naked eye or in non-controlled circumstances, as mentioned above, with the use of platforms coupled to smartphones, it is possible to decrease variation between determinations [26,84]. Sample pretreatment is fundamental to most analytical methods, so it is essential to consider that paper-based POC platforms are no exception. The methods should be able to detect the signal robustly despite noise or include in situ pretreatment if necessary, and less complex matrices are recommended when possible, such as the use of serum instead of whole blood [40,140]. In the case of devices for detecting nucleic acids, in some cases, they require pretreatment steps of the sample for the extraction or even amplification of the genetic material [130]. In addition, since they are a miniaturized platform, paper-based POCs restrict the sample volume that can be analyzed, which adds a constraint on the sensitivity and detection limit [141,142]. The use of sophisticated fabrication techniques increases the performance of the generated POC platforms. It allows the generation of robust multiplex platforms but with the limitation of increasing their cost and decreasing the possibility of applying them in low-cost setups. Despite the drawbacks mentioned above, the demonstrated potential of paper-based POC platforms ensures that their study continues to be a growing area of research.

New devices must be designed from the early stages with a complete vision of the needs they are intended to meet and the context in which they are intended to be used. Otherwise, the prototypes will present problems at the design level that will prevent them from even reaching the clinical testing stage [135,142]. To achieve the goal of an ideal platform, a balance must be struck between design complexity and feasibility of implementation, especially in remote settings, and for this alternative to be attractive to the market, optimization times and mass production costs must be taken into account. Considering the massive boost that the commercialization of pregnancy testing gave to the development of lateral fluid devices, it is feasible that the successful market introduction of a paper-based device for the diagnosis and monitoring of T2DM will spur the development of other similar platforms [50]. Despite numerous studies reporting the successful determination of glucose using paper-based platforms, these have not been successfully translated to the market. One of the main limitations is the regulatory barriers and technical challenges that still prevent their mass production.

**Figure 3:**
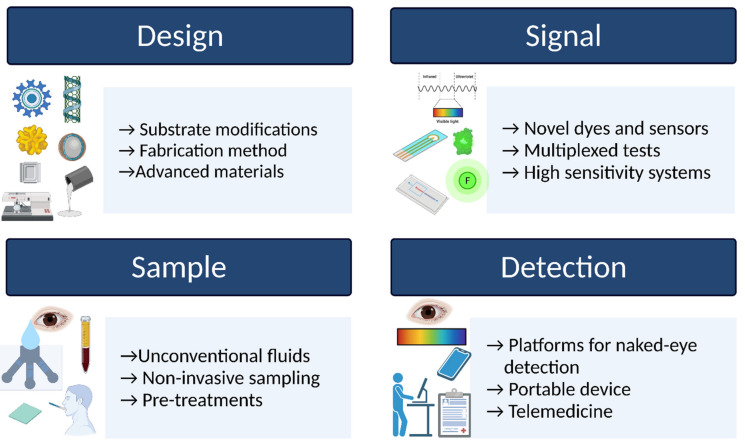
Opportunity and improvement areas for the development of paper-based platforms for type 2 diabetes diagnosis and monitoring. Created with BioRender.

Another limitation in the implementation of paper-based POCs is the interpretation of results; even in the case of glucometers that have been widely used for decades, the data must sometimes be interpreted by the physician or other trained personnel to make health decisions. This creates a constraint in remote and low-resource settings, diminishing the cost, speed, and accessibility advantages of paper-based POCs. An alternative to overcome this obstacle is the use of telemedicine, which allows access to health services in remote or low-resource settings [13,27,50]. Another option is the development of applications that allow self-monitoring with artificial intelligence for data processing. The applications indicate relevant information to the patient on a smartphone and store a patient record that a physician can access through an internet cloud service. Additionally, accessibility, costs, and connectivity issues must be considered. However, for some applications, such as the maintenance of blood glucose levels, it would be possible to include a color guide for qualitative interpretation with the naked eye, or smartphone applications can be developed to detect and interpret results based on standards that eliminate variations due to illumination and other environmental factors [13,27,50]. Although enzymatic paper-based POC platforms have demonstrated their potential in the diagnostic area, there are still areas of opportunity and improvement to enable their mass production and market entry. An overview of the main areas in terms of design, signal acquisition, sampling, and detection is summarized in Figure 3. The development of diagnostic POCs on a commercial level requires a significant initial investment, and to achieve this investment, it must demonstrate significant market potential [11]. This is more feasible in fast-growing markets, and those with recognized unmet needs such as intermediate-term blood glucose monitoring. Despite evidence that lifestyle changes can prevent the development of T2DM and that maintenance of glycemic control decreases the risk of complications, there is a reluctance in the general population to make these lifestyle changes, sometimes because they do not perceive themselves to be at risk for T2DM or do not have access to adequate health information and resources. Having accessible and cost-effective tools for self-monitoring will foster the culture of self-care. In this sense, paper-based POC platforms are highly relevant alternatives due to their unique capabilities and functionalities. Bringing these types of POC devices to the general population in conjunction with health information and mass screening campaigns can positively impact the prevention and early diagnosis of T2DM. 

## Figures and Tables

**Table 1:** Summary of reported enzymatic paper-based platforms in representative references.

Substrate	System	Sample	Detection	LOD (mg/dL)	References
Wax printing on Whatman chromatography paper 595	GOD/HRP/ 4-AAP/HBA	Tears	Smartphone camera	NM	[74]
Whatman filter paper No. 1 with lamination film	GOD/BP	Saliva	Smartphone camera	24.6	[75]
Wax printing on qualitative filter paper and Schirmer strips	GOD/Au(I) complex (AuC_2_C_6_H_4_OMe)_2_ (Ph_2_P(C_6_H4)_3_PPh_2_)	Simulated tear fluid and blood	Bifurcated optical fiber system	16.2 (plasma) 1.4 (tear)	[79]
Whatman cellulose filter paper No. 1 treated with CH	GOD/HRP/ EDC/*o*-PD	Urine	Smartphone camera	18.0	[80]
Whatman filter paper No. 40 stamped with paraffin and treated with CH	GOD/HRP/ TBHBA/4-AAP	Artificial and human saliva	Naked eye	0.8	[76]
High-purity cellulose membranes	GOD/HRP/TMB	Urine	Digital camera	8.1	[69]
Whatman filter paper No. 1 with lamination film	GOD/BP	Saliva	Handheld optical biosensor	32.0	[81]
Wax printing on Whatman filter paper No. 1	GOD/HRP/ KI or TMB	Plasma	Smartphone camera	27.0 (KI) 0.9 (TMB)	[82]
Wax printing on Whatman filter paper No. 1 treated with CH	GOD/HRP/ 4-AAP/HBA	Blood	Scanner	NM	[70]
Whatman qualitative paper No.1 treated with PB	GOD	Serum	Distance-based measurements	19.8	[83]
Nitrocellulose membranes	GOD/HRP/4-AAP/COL/MADB	Serum	Chemidoc imaging system	0.2	[71]
Wax printing on Whatman No. 1 cellulose chromatography paper treated with BSA	GOD/HRP/ 4-AAP/DHBS	Serum	Scanner	5.4	[4]
Whatman qualitative filter paper No. 1 coated with a UV-curable resin	GOD/HRP/ MAOS/4-AAP	Serum	Smartphone camera	5.4	[72]
Wax printing in Whatman No. 1 chromatography filter paper treated with CH	GOD/HRP/TMB	Blood	Smartphone-based optical platform	5.0	[84]
Whatman filter paper No. 3 treated with OTS and MTS	GOD/HRP/ phenol/4-AAP	Plasma	Portable scanner	15.1	[45]
Wax printing in Whatman No. 1 qualitative filter paper loaded with ZnNR	GOD/ 4-AAP/ DHBS	Serum and urine	Smartphone camera	0.05	[73]
Whatman filter paper No. 41 treated with BSA-Tween	GOD/HRP/TMB	Sweat	Scanner and Smartphone camera	0.18	[77]

4-AAP: 4-amino antipyrine; BP: bromocresol purple; CH: chitosan; BSA: Bovine serum albumin; COL: chitosan oligosaccharide lactate; DHBS: 3,5-dichloro-2-hydroxy acid sodium; EDC: N-(3-Dimethylaminopropyl)-N′-ethylcarbodiimide hydrochloride; GOD: Glucose oxidase; HBA: 4-Hydroxybenzoic acid; HRP: Horseradish peroxidase; LOD: Limit of detection; MADB: N, N-Bis(4-sulfobutyl)-3,5-dimethylaniline disodium salt; MAOS: N-Ethyl-N-(2-hydroxy-3-sulfopropyl)-3,5-dimethylaniline sodium salt monohydrate; MTS: methyltrichlorosilane; *o*-PD: *o*-phenylenediamine; NM: not mentioned; TBHBA: 2,4,6-tribromo-3-hydroxy benzoic acid; OTS: octadecyltrichlorosilane; PB: Prussian blue; TMB: 3,3’,5,5’-tetramethylbenzidine; ZnNR: zinc oxide nanorods.
